# Multi-Locus Variable Number of Tandem Repeat Analysis for Rapid and Accurate Typing of Virulent Multidrug Resistant *Escherichia coli* Clones

**DOI:** 10.1371/journal.pone.0041232

**Published:** 2012-07-27

**Authors:** Umaer Naseer, Barbro E. Olsson-Liljequist, Neil Woodford, Hiran Dhanji, Rafael Cantón, Arnfinn Sundsfjord, Bjørn-Arne Lindstedt

**Affiliations:** 1 Research Group for Host-Microbe Interactions, Department of Medical Biology, University of Tromsø, Tromsø, Norway; 2 Unit of Antibiotics and Infection Control, Swedish Institute for Communicable Disease Control, Solna, Sweden; 3 Antibiotic Resistance Monitoring and Reference Laboratory, Health Protection Agency, London, United Kingdom; 4 Servicio de Microbiología and CIBER en Epidemiología y Salud Pública (CIBERESP), Instituto Ramón y Cajal de Investigación Sanitaria (IRYCIS) and Hospital Universitario Ramón y Cajal, Madrid, Spain; 5 Unidad de Resistencia a Antibióticos y Virulencia Bacteriana asociada al Consejo Superior de Investigaciones Científicas (CSIC), Madrid, Spain; 6 Reference Centre for Detection of Antimicrobial Resistance, Department of Microbiology and Infection Control, University Hospital of North Norway, Tromsø, Norway; 7 Division of Infectious Diseases control, Norwegian Institute of Public Health, Oslo, Norway; Charité-University Medicine Berlin, Germany

## Abstract

One hundred *E. coli* isolates from Norway (n = 37), Sweden (n = 24), UK (n = 20) and Spain (n = 19), producing CTX-M-type - (n = 84), or SHV-12 (n = 4) extended spectrum β-lactamases, or the plasmid mediated AmpC, CMY-2 (n = 12), were typed using multi-locus sequence typing (MLST) and multi-locus variable number of tandem repeat analysis (MLVA). Isolates clustered into 33 Sequence Types (STs) and 14 Sequence Type Complexes (STCs), and 58 MLVA-Types (MTs) and 25 different MLVA-Type Complexes (MTCs). A strong agreement between the MLST profile and MLVA typing results was observed, in which all ST131-isolates (n = 39) and most of the STC-648 (n = 10), STC-38 (n = 9), STC-10 (n = 9), STC-405 (n = 8) and STC-23 (n = 6) isolates were clustered distinctly into MTC-29, -36, -20, -14, -10 and -39, respectively. MLVA is a rapid and accurate tool for genotyping isolates of globally disseminated virulent multidrug resistant *E. coli* lineages, including ST131.

## Introduction

Antibiotic resistance is a globally interrelated public health problem, the threat of which is ever increasing. Dispersion of large mobile genetic elements carrying multiple resistance determinants coupled with dissemination of successful clones have been chronicled in most bacterial species [1], [2]. Locally, antibiotic exposure selects and promotes the evolution of multidrug resistant (MDR) bacteria, [3], [4] and movement of people and transport of food facilitate their worldwide dispersion [5]. MDR bacteria both undermine empirical treatment and delay appropriate therapy, which in turn increases patient mortality [6]. Therefore preventing the spread of MDR bacteria is an infection control priority [2]. An integral part of infection control is the recognition of successful and emerging MDR strains or clones, and hence there is always a need for rapid and accurate typing tools.

β-lactams belong to our most important and widely used class of antibiotics. Bacteria belonging to the Enterobacteriaceae family have during the last decades evolved to enable carriage of multiple β-lactamases, including extended-spectrum β-lactamases (ESBLs), plasmid mediated AmpC enzymes as well as carbapenemases such as VIM, NDM, KPC and OXA-48, which has compromised the clinical utility of β-lactam drugs [7]. In particular, Enterobacteriaceae strains carrying CTX-M type ESBLs have become prevalent and dominant worldwide [8], [9], [10], and clonal spread of CTX-M producing *E. coli* co-resistant to trimethoprim-sulfamethoxazole and ciprofloxacin are increasingly reported [11]. The worldwide emergence of the clone O25:H4 - ST131 as a dominant host of ESBLs poses huge public health challenges, and never have there been a greater need for a rapid detection and an intercontinental monitoring system for these clones [12], [13].

ST131 was originally identified using the Achtman multi-locus sequence typing (MLST) scheme [14], but faster methods, specific for the detection of ST131 have also been reported, including commercial repetitive sequence-based PCR (rep-PCR) [15], single nucleotide polymorphism (SNP) analysis of *mdh* and *gyrB* combined with O25b *rfb* [16], PCR approach using O25-*pabB* [17], and a ST131 CTX-M-15 specific triplex PCR for operon afa FM95545 [18].

Several studies have explored the use of tandem repeated DNA as mean for identification of different bacterial strains. Multi-locus variable number of tandem repeat analysis (MLVA) has been successfully applied for rapid and interlaboratory typing of various bacteria such as *Bacillus*, *Yersinia*, *Mycobacterium*, *Enterococcus*, *Staphylococcus*, *Neisseria, Salmonella* and *E. coli* O157 [19]. In this study we explore the use of MLVA as a possible typing tool for different globally dispersed virulent multidrug resistant lineages of *E. coli*.

## Materials and Methods

List of abbreviations and resistance genes given in Table 1.

**Table 1:** List of abbreviations and resistance genes.

*bla*		β-lactamase
	CTX-M	Cefotaximase - Munich
	SHV	Sulfhydryl reagent variable
	CMY	Cephamycinase
	VIM	Verona imipenemase
	NDM	New Delhi metallo β-lactamase
	KPC	Klebsiella pneumoniae carbapenemase
	OXA	Oxacillinase
MLST		Multi-locus sequence typing
ST		Sequence type
STC		Sequence type complex
MLVA		Multi-locus variable number of tandem repeat analysis
MT		MLVA type
MTC		MLVA type complex
SLV		Single locus variant
DLV		Double locus variant
TLV		Triple locus variant
QLV		Quadruple locus variant

### Bacterial Strains

**Figure 1:**
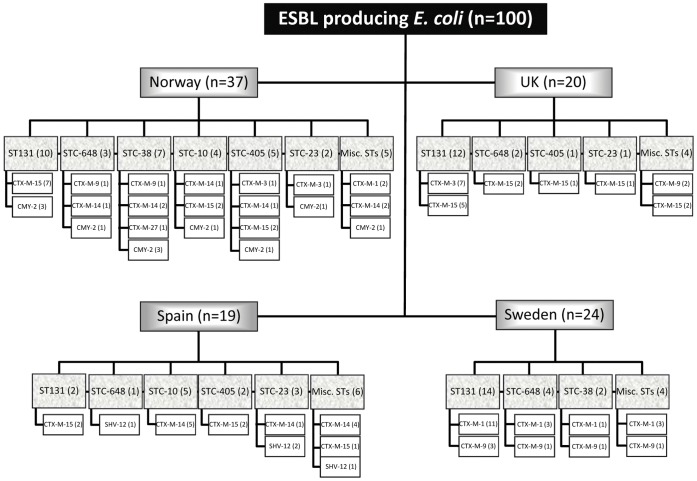
Overview of the 100 *E. coli* isolates included in this study from the four participating countries, their sequence types and genotypes.

A total of 100 well-characterised clinical *E. coli* isolates producing ESBLs- or plasmid-mediated AmpC were tested. They had been collected between year 2000–2008, from Norway (n = 37), Sweden (n = 24), UK (n = 20) and Spain (n = 19), and of these, 84 isolates were CTX-M producers [CTX-M-1 (n = 20), CTX-M-3 (n = 9), CTX-M-15 (n = 27), CTX-M-27 (n = 1), CTX-M-9 (n = 10) and CTX-M-14 (n = 17)], 12 were CMY-2 producers and 4 were SHV-12 producers (Figure 1).

### Multi-locus Sequence Typing (MLST)

MLST was performed according to the scheme developed by Achtmans group targeting seven housekeeping genes; *adk*, *fumC*, *gyrB*, *icd*, *mdh*, *purA*, and *recA*, Target genes were amplified and sequenced using primers and conditions described on the web-site (http://mlst.ucc.ie/) [14]. Furthermore, allele numbers were assigned; Sequence Types (ST) were generated and assigned to the different Sequence Type Complexes (STCs) using the web-site from the respective laboratories. Collected MLST data were entered into BioNumerics (Applied Maths, Foster City, California) for comparative analysis.

### Multi-locus Variable Number of Tandem Repeat Analysis (MLVA)

Total DNA of *E. coli* was prepared from overnight cultures using the RT Spin Bacteria DNA minikit (Invitek, Berlin, Germany). MLVA was performed in a single laboratory by targeting nine tandem repeats (CVN001, CVN002, CVN003, CVN004, CVN007, CVN014, CVN015, CCR001 and CVN016) identified and described by Lindstedt et al. [20], [21]. Repeats were amplified using PCR and multiple flour-labelled primers discernible by a genetic analyser. PCR products were subjected to capillary electrophoresis on an ABI-3130 Genetic Analyzer (Applied Biosystems, Oslo, Norway). Each peak was identified according to colour and size, and an allele number was assigned based on fragment sizes. Alleles for which amplicons were absent were designated an allele number of ‘0’. The allele numbers were entered into BioNumerics as character values and a dendrogram was constructed using categorical coefficients and the Ward algorithm. Numerical values were assigned for distinct MLVA-type profiles (MTs) and MLVA-Type Complexes (MTCs) were assigned for related isolates of up to four locus variations.

## Results

### Multi-locus Sequence Typing (MLST)

The 100 isolates were typed to 33 different Sequence Types (STs) and 14 different Sequence Type Complexes (STCs). A total of 81 of the 100 isolates were identified from 6 globally dispersed ST or STCs; i) ST131 (n = 39), ii) STC-648 (n = 10) spanning 3 different STs, iii) STC-38 (n = 9), spanning 2 different STs, iv) STC-10 (n = 9), spanning 4 different STs, v) STC-405 (n = 8), spanning 2 different STs, and vi) STC-23 (n = 6), spanning 4 different STs (Table 2). The remaining 19 isolates had 18 different STs which clustered into 9 different STCs (Table S1).

**Table 2:** Six major sequence type complexes (STCs) and sequence types (STs) in relation to assigned MLVA-types (MTs) and MLVA-type complexes (MTCs).

STC	ST	No. (n) Isolates	*bla*	MT	MTC	Locus variation
**None**	131	11	CTX-M-15	6-0-0-10-3-4-1-6-0	29	
	131	7	CTX-M-3	6-0-0-10-3-4-1-6-0	29	
	131	4	CTX-M-1	6-0-0-10-3-4-1-6-0	29	
	131	1	CTX-M-9	6-0-0-10-3-4-1-6-0	29	
	131	2	CTX-M-1	6-0-0-10-3-8-1-6-0	29	SLV 29 (CVN014)
	131	2	CTX-M-15	6-0-0-10-3-3-1-6-0	29	SLV 29 (CVN014)
	131	1	CTX-M-1	6-0-0-10-2-4-1-6-0	29	SLV 29 (CVN014)
	131	1	CTX-M-1	6-0-0-10-3-11-1-6-0	29	SLV 29 (CVN014)
	131	1	CTX-M-1	6-0-0-10-3-7-1-6-0	29	SLV 29 (CVN014)
	131	1	CTX-M-9	6-0-0-10-3-10-1-6-0	29	SLV 29 (CVN014)
	131	1	CTX-M-15	6-0-0-10-3-2-1-6-0	29	SLV 29 (CVN014)
	131	1	CMY-2	6-0-0-10-3-5-1-6-0	29	SLV 29 (CVN014)
	131	1	CTX-M-1	2-0-0-10-3-4-1-6-0	29	SLV 29 (CVN001)
	131	1	CMY-2	6-3-0-10-3-4-1-6-0	29	SLV 29 (CVN002)
	131	1	CTX-M-1	6-0-0-6-3-4-1-6-0	29	SLV 29 (CVN004)
	131	1	CTX-M-9	6-0-0-10-3-6-1-39-0	29	DLV 29 (CVN014, CCR001)
	131	1	CMY-2	5-0-0-10-3-5-1-0-41	29	TLV 29 (CVN001, CVN014, CCR001)
	131	1	CMY-2	6-3-0-8-3-5-1-6-7	29	QLV 29 (CVN002, CVN004, CVN014, CVN016)
**648**	648	2	CTX-M-1	6-0-0-8-2-8-1-16-6	36	
	648	1	CTX-M-15	6-0-0-8-2-8-1-16-6	36	
	648	1	CMY-2	6-3-0-8-2-8-1-16-6	36	SLV 36 (CVN002)
	648	1	CTX-M-9	6-0-0-8-2-5-1-16-6	36	SLV 36 (CVN014)
	648	1	CTX-M-9	6-1-0-8-2-7-1-16-6	36	DLV 36 (CVN002, CVN014)
	648	1	CTX-M-1	6-18-0-8-2-7-1-16-6	36	DLV 36 (CVN002, CVN014)
	648	1	SHV-12	6-3-0-8-2-7-1-16-6	36	DLV 36 (CVN002, CVN014)
	684	1	CTX-M-15	5-0-7-0-3-1-1-6-0	6	
	62	1	CTX-M-14	6-0-0-10-3-4-1-6-0	29	
**38**	38	2	CTX-M-14	5-3-0-10-3-8-1-64-20	20	
	38	1	CTX-M-9	5-3-0-10-3-8-1-64-20	20	
	38	1	CMY-2	5-3-0-10-3-8-1-64-17	20	SLV 20 (CVN016)
	38	1	CTX-M-1	5-3-0-10-3-7-1-64-15	20	DLV 20 (CVN014, CVN016)
	38	1	CTX-M-9	5-3-0-10-3-4-1-64-10	20	DLV 20 (CVN014, CVN016)
	38	1	CMY-2	5-3-0-10-3-7-1-64-17	20	DLV 20 (CVN014, CVN016)
	38	1	CTX-M-27	5-3-0-8-3-8-1-55-17	20	TLV 20 (CVN004, CCR001, CVN016)
	778	1	CMY-2	6-0-0-10-3-10-1-6-0	25	
**10**	167	2	CTX-M-15	5-10-7-8-3-1-1-6-7	14	
	167	1	CTX-M-14	5-10-7-8-3-1-1-6-7	14	
	167	1	CTX-M-14	5-8-8-8-4-1-1-6-7	14	TLV 14 (CVN002, CVN003, CVN007)
	617	1	CTX-M-14	5-10-7-8-3-1-1-6-7	14	
	10	1	CTX-M-14	5-0-7-8-3-3-1-6-7	14	DLV 14 (CVN002, CVN014)
	10	1	CMY-2	5-1-5-8-3-1-1-6-8	14	TLV 14 (CVN002, CVN003, CVN016)
	10	1	CTX-M-14	5-0-0-8-3-5-1-35-0	5	
	48	1	CTX-M-14	5-0-0-8-3-5-1-35-0	5	
**405**	405	1	CMY-2	5-1-0-10-3-4-1-0-47	10	
	405	1	CTX-M-15	5-1-0-10-3-4-1-0-47	10	
	405	1	CTX-M-15	5-1-0-10-3-4-1-0-44	10	SLV 10 (CVN016)
	405	1	CTX-M-3	5-1-0-10-3-4-1-64-0	10	DLV 10 (CCR001, CVN016)
	405	1	CTX-M-14	5-1-0-10-3-5-1-64-21	10	TLV 10 (CVN014, CCR001, CVN016)
	964	2	CTX-M-15	5-1-0-10-3-4-1-64-24	10	DLV 10 (CCR001, CVN016)
	405	1	CTX-M-15	5-3-0-9-3-3-1-95-10	22	
**23**	23	1	SHV-12	6-0-0-8-3-5-1-6-0	39	
	410	1	CTX-M-15	6-0-0-8-3-5-1-6-0	39	
	88	1	SHV-12	6-3-0-8-3-15-1-6-0	39	DLV 39 (CVN002, CVN014)
	90	1	CTX-M-14	6-1-0-8-3-3-1-6-7	39	TLV 39 (CVN002, CVN014, CVN016)
	90	1	CTX-M-3	6-1-0-8-3-4-1-6-7	39	TLV 39 (CVN002, CVN014, CVN016)
	88	1	CMY-2	7-0-0-3-1-7-1-16-0	55	

* For complete data on all 100 isolates see Table S1.

### Multi-locus Variable Number of Tandem Repeat Analysis (MLVA)

The 100 isolates were typed to 58 different MLVA-types (MTs) and 25 different MLVA Complexes (MTCs) of related isolates. Isolates belonging to the six major STs or STCs were clustered as; i) ST131 (n = 39/39) and ST62 (n = 1/1) -isolates into MTC labelled 29, spanning 14 MTs, ii) STC-648 (n = 8/10) into MTC labelled 36, spanning 6 MTs, iii) STC-38 (n = 8/9) into MTC labelled 20, spanning 6 MTs, iv) STC-10 (n = 7/9) into MTC labelled 14, spanning 4 MTs v) STC-405 (n = 7/8) into MTC labelled 10, spanning 5 MTs, and vi) STC-23 (n = 5/6) into MTC labelled 39, spanning 4 MTs (Table 2). The remaining 26 isolates were distributed into 19 MTs (Table S1).

### MLST and MLVA by Country and Genotypes

ST131 isolates (n = 39) were collected from Sweden (n = 14), UK (n = 12), Norway (n = 11), and Spain (n = 2), encoding β-lactamases CTX-M-1 (n = 11), CTX-M-3 (n = 7), CTX-M-15 (n = 14), CTX-M-9 (n = 3), and CMY-2 (n = 4). All 39 isolates were typed into a single MTC-29, with 24 identical isolates labelled MT29, 12 SLVs, 1 DLV, 1 TLV and 1 QLV (Table 2). Interestingly, 86% (n = 18/21) of the ST131 isolates with CTX-M-15 and its precursor CTX-M-3 producing isolates (all from the UK), had an indistinguishable MLVA genotype, MT29. Whereas greater MLVA diversity was seen among ST131 isolates with CTX-M-9 and CMY-2 enzymes.

STC-648 isolates (n = 10) were collected from Sweden (n = 4), Norway (n = 3), UK (n = 2) and Spain (n = 1), encoding β-lactamases SHV-12 (n = 1), CTX-M-1 (n = 3), CTX-M-15 (n = 2), CTX-M-9 (n = 2), CTX-M-14 (n = 1), and CMY-2 (n = 1). The majority of the strains (n = 8) were grouped into a single MTC-36, consisting of six different MTs, varying exclusively at loci CVN002 and/or CVN014.

The STC-38 isolates (n = 9) were collected only from Norway (n = 7) and Sweden (n = 2), encoding β-lactamases CTX-M-1 (n = 1), CTX-M-9 (n = 2), CTX-M-14 (n = 2), CTX-M-27 (n = 1) and CMY-2 (n = 3). Eight of these isolates were typed into MTC-20, consisting of six different MTs, varying at loci CVN014 and/or CVN016. All CTX-M-9 and CTX-M-14 isolates from Norway were grouped into a single genotype MT20.

Isolates belonging to the STC-405 (n = 8) were collected from Norway (n = 5), Spain (n = 2) and UK (n = 1), encoding β-lactamases CTX-M-3 (n = 1), CTX-M-15 (n = 5), CTX-M-14 (n = 1) and CMY-2 (n = 1). Seven of the isolates were grouped into MTC-10, consisting of five different MTs, mainly varying at loci CCR001 and CVN016.

The STC-10 isolates (n = 9) were collected from Spain (n = 5) and Norway (n = 4), encoding β-lactamases CTX-M-14 (n = 6), CTX-M-15 (n = 2) and CMY-2 (n = 1). Seven of these isolates were typed into MTC-14, and the remaining two CTX-M-14 producing isolates from Spain in MTC-5. The MTC-14 was consisting of four different MTs. Six isolates were collected from Spain (n = 3), Norway (n = 2) and UK (n = 1), encoding β-lactamases SHV-12 (n = 2), CTX-M-3 (n = 1), CTX-M-15 (n = 1), CTX-M-14 (n = 1), and CMY-2 (n = 1) of the STC-23. Five of the isolates were grouped into a single MTC-39, varying at loci CVN002, CVN014 and CVN016.

## Discussion

**Figure 2:**
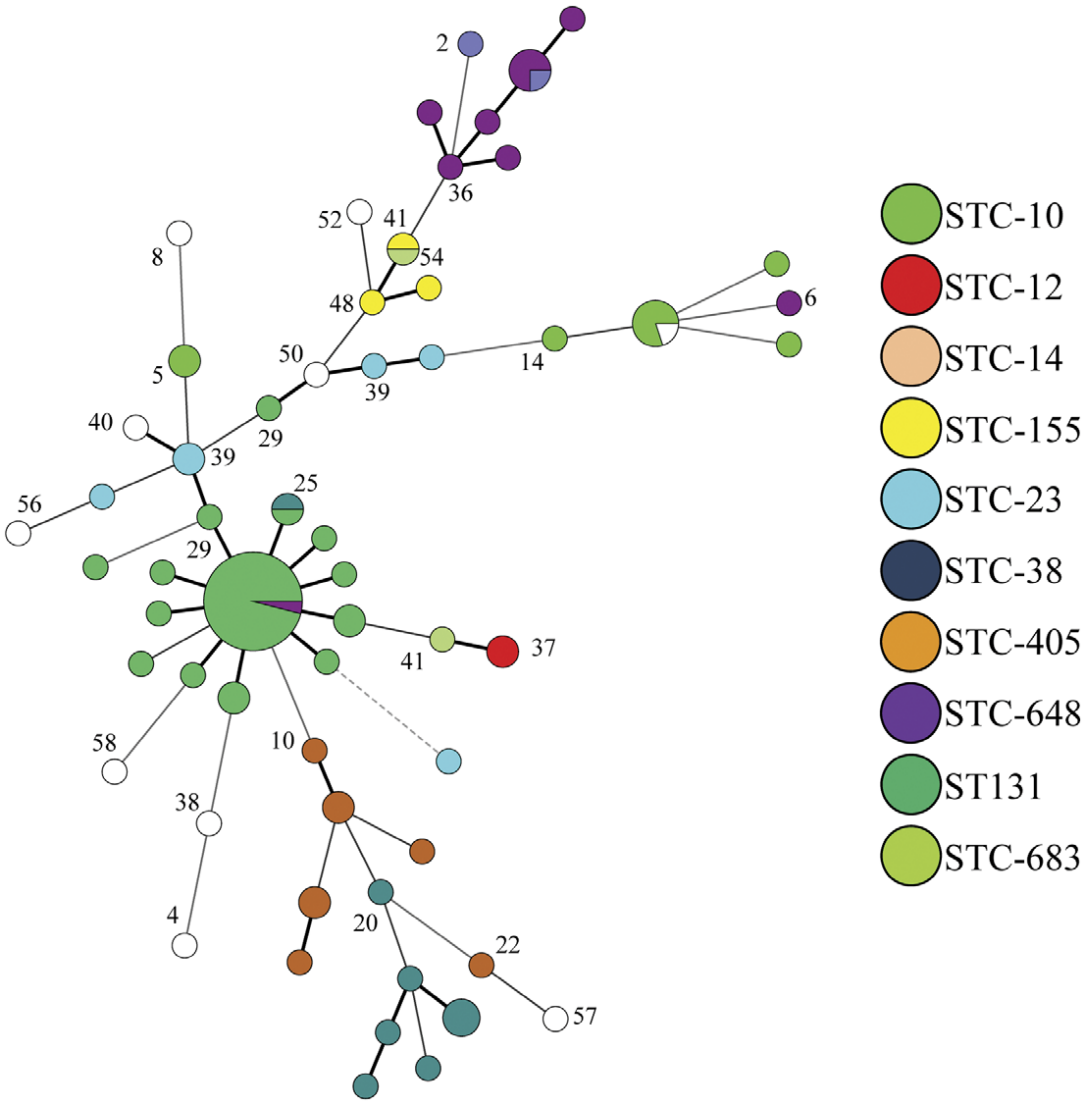
Minimum-spanning tree analysis of MLVA data from 100 *E. coli* isolates. The circles represent unique MLVA types (numeric values); the diameter of the circles represents number of isolates. MLST data on the strains are color-coded: STC-10 (fluorescent green), STC-12 (red), STC-14 (dark blue), STC-155 (yellow), STC-23 (light blue) STC-38 (dark green), STC-405 (brown), STC-648 (purple), ST131 (green), STC-683 (light green).

In this study we document MLVA as a tool for rapid and accurate typing of internationally-dispersed virulent ESBL producing *E. coli* lineages, including ST131. Independently collected isolates from four countries during 9 years displayed high concordance between MLST types and MLVA types with an increased resolution (Figure 2). Interestingly, the MLVA typing scheme was in particular able to genotype the six major STs and STCs responsible for the pandemic spread of ESBLs, into six distinctly different MTCs. No country-specific clustering of isolates was seen. The variability within the nine different loci under investigation suggested that the present MLVA scheme and its combination of highly variable and conservative loci can accurately serve the purpose of genotyping these strains. We recognized that in our collection, locus CVN015 had none, and loci CVN003 and CVN007 had little discriminatory power. CVN003 was only present in nine isolates with three different alleles, and CVN007 was identified with only a single allele 3 in 87% of the isolates. Nevertheless these loci have in the past proven to be important markers for genotyping enteropathogenic *E. coli* of diverse serotypes [21]. It is here also interesting to highlight that alleles 10 and 8 at locus CVN004 seem to be conserved (93%) across all isolates of the major STs and STCs, and in an MLVA database of *E. coli* isolates (n = 3417), allele 10 at locus CVN004 is only present in 4% of the isolates (n = 139) including the strains from this study (personal database of Lindstedt). Thus, the particular alleles 8 and 10, at locus CVN004 seem to be important indicators for the presence of the major STCs and ST131.

Currently, phenotypic detection of the ST131 clone is not possible and molecular-based techniques are required. Accurate detection is an important first step towards monitoring and controlling ST131 dissemination. ST131 *E. coli* producing CTX-M-15 are usually co-resistant to quinolones and aminoglycosides [22], [23], which leaves few therapeutic options against infections in human or animal [24], [25]. In recent years, NDM-1 and KPC-2 carbapenemase producing ST131 has also been reported, substantiating the need for a rapid detection method of this clone [26], [27]. Indeed, newer, faster and more comprehensive typing technology is being developed and introduced. In wait of full genome sequencing as an affordable and manageable tool for typing, promising alternative typing techniques for *E. coli* in addition to MLVA include DNA microarray [28], [29] and Matrix-assisted laser desorption ionization-time of flight mass spectrometry, MALDI-TOF-MS [30]. Although these techniques are initially developed for accurate and rapid typing of specific pathogenic *E. coli* serotypes, they hold the potential for also typing MDR lineages of *E. coli.* Indeed, as we have successfully shown with the existing MLVA scheme; a potential for rapid, accurate and high resolution genotyping of ST131 and other internationally-dispersed, virulent multidrug resistant *E. coli* lineages.

We have identified MLVA type complexes that correspond to the major MLST-defined complexes. Assuming extracted DNA and availability of all required equipment, the expected turnover time for MLVA of a single isolate is close to five hours, whereas it may require a couple of days for its complete MLST analysis. As such MLVA is a good alternative to MLST for epidemiological surveillance of virulent multidrug resistant *E. coli* in the future [2].

## Supporting Information

Table S1 **MLST and MLVA data on all 100 isolates included in this study.**
(XLSX)
